# The Effects of Java Water-Dropwort (*Oenanthe javanica*) Extract on Alcohol Metabolism and Hangover Symptoms: A Randomized, Double-Blind, Placebo-Controlled Crossover Clinical Trial

**DOI:** 10.3390/foods15061003

**Published:** 2026-03-12

**Authors:** Young-Sik Kim, Chan-Hun Jung, Seon-Young Kim, Hyungyung Chai, Hongjun Kim

**Affiliations:** 1 Department of Herbology, College of Korean Medicine, Woosuk University, Jeonju 55338, Republic of Korea; yjbsik@woosuk.ac.kr; 2 Jeonju AgroBio-Materials Institute, Wonjangdong-gil 111-27, Jeonju 54810, Republic of Korea; biohun@gmail.com (C.-H.J.); seon02@jami.re.kr (S.-Y.K.); 3 Research Institute, MediCRO Co., Ltd., Anyang 14067, Republic of Korea; biostat@medicro.co.kr; 4 Department of Formula Science, College of Korean Medicine, Woosuk University, Jeonju 55338, Republic of Korea

**Keywords:** java water-dropwort, *Oenanthe javanica*, hangover, alcohol metabolism, acetaldehyde, clinical trial

## Abstract

Alcohol hangover is a significant health concern worldwide. Java water-dropwort (*Oenanthe javanica*) has been traditionally used in East Asia for treating hepatitis, jaundice, and alcohol hangovers. This study evaluated the effects of java water-dropwort extract formulation on alcohol metabolism and hangover symptoms. A randomized, double-blind, placebo-controlled crossover trial was conducted with 36 healthy adults aged 19–40 years. Participants received either java water-dropwort extract formulation (8.71 mL equivalent to 6.69 g raw material) or placebo 30 min before alcohol consumption (0.8 g/kg body weight, 20.1% soju). Blood alcohol and acetaldehyde concentrations were measured at multiple time points up to 15 h post consumption. Hangover symptoms were assessed using the Alcohol Hangover Severity Scale and Acute Hangover Scale. A total of 36 participants were enrolled, and 33 completed the study per protocol. Blood alcohol area under the curve (AUC) was significantly lower in the java water-dropwort group (58.626 vs. 66.194 mmol·h/L, *p* = 0.008). Blood acetaldehyde AUC was also significantly reduced (69.794 vs. 88.205 mg·h/dL, *p* = 0.031). Hangover symptom scores in the test group were significantly lower than those in the placebo group (4.030 vs. 8.606, *p* = 0.026). No adverse events occurred. Java water-dropwort extract effectively enhanced alcohol metabolism and improved hangover symptoms, offering potential therapeutic value for hangover management.

## 1. Introduction

Alcohol hangover, characterized by a constellation of unpleasant physical and psychological symptoms following excessive alcohol consumption, is a common experience among alcohol consumers, with hangover frequency strongly correlating with severity [1]. The primary symptoms include headache, nausea, fatigue, dizziness, and gastrointestinal disturbances, significantly impacting quality of life and productivity [2]. The economic burden of hangover-related absenteeism and reduced workplace performance is substantial, highlighting the need for effective interventions [3].

The pathophysiology of alcohol hangover is multifactorial, involving alcohol metabolism, acetaldehyde accumulation, dehydration, electrolyte imbalance, inflammatory responses, and oxidative stress. Ethanol is primarily metabolized in the liver by alcohol dehydrogenase (ADH) to acetaldehyde, which is subsequently oxidized to acetate by aldehyde dehydrogenase (ALDH). Acetaldehyde, being highly toxic, is considered a major contributor to hangover symptoms when accumulated in the blood [4]. Therefore, interventions that enhance alcohol metabolism and accelerate acetaldehyde clearance may potentially alleviate hangover symptoms [5].

Java water-dropwort (*Oenanthe javanica* DC.), a perennial aquatic plant belonging to the Apiaceae family, has been traditionally used in East Asian countries, including Korea, China, and Japan, for various medicinal purposes, including alcohol detoxification [6,7]. The plant is rich in phenolic compounds, flavonoids, vitamins, and minerals, exhibiting antioxidant, hepatoprotective, and anti-inflammatory properties [8]. Previous studies have demonstrated that java water-dropwort extracts possess hepatoprotective effects against alcohol-induced liver damage and may enhance alcohol metabolism in animal models [9,10].

Recent clinical trials have investigated various natural compounds for hangover relief, showing promising results with herbal extracts that enhance alcohol metabolism through ADH and ALDH activation [11,12]. However, limited clinical evidence exists regarding the efficacy of java water-dropwort extract in human subjects for hangover management. Given the traditional use and preclinical evidence supporting its potential benefits, a rigorous clinical evaluation of java water-dropwort extract’s effects on alcohol metabolism and hangover symptoms is warranted.

This study aimed to evaluate the efficacy and safety of a java water-dropwort extract-containing formulation in improving alcohol metabolism and reducing hangover symptoms in healthy adults through a randomized, double-blind, placebo-controlled crossover clinical trial.

## 2. Materials and Methods

### 2.1. Study Design and Ethics

This study was conducted as a randomized, double-blind, placebo-controlled crossover clinical trial at Bestian Seoul Hospital, Seoul, South Korea. In South Korea, human application tests for health functional foods are exempt from mandatory prior clinical trial registration or authorization by the Ministry of Food and Drug Safety (MFDS), which is distinct from pharmaceutical clinical trials. According to the MFDS ‘Guideline for Designing Human Application Tests for Developers of Health Functional Foods’ and the ‘Regulation on Recognition of Functional Ingredient and Standard/Specification for Health Functional Foods’ (Article 14, Paragraph 8, Item C), such studies must be conducted in accordance with the International Conference on Harmonization Good Clinical Practice (ICH GCP) guidelines and require approval from an institutional review board (IRB) [13]. Accordingly, the study protocol was approved by the Institutional Review Board of Bestian Seoul Hospital (IRB approval number: 2024-07-006) and conducted in accordance with the Declaration of Helsinki and Good Clinical Practice guidelines. All participants provided written informed consent before enrollment.

### 2.2. Participants

Healthy adults aged 19–40 years who had experienced hangover symptoms after alcohol consumption within the previous month were recruited. The sample size was calculated based on blood alcohol concentration area under the curve (AUC) as the primary endpoint, with an expected difference of 196.8 mg·h/L between groups, assuming α = 0.05, β = 0.2, and a 30% dropout rate, resulting in a target enrollment of 36 participants.

Inclusion criteria: (1) healthy adults aged 19–40 years; (2) BMI 18.5–30.0 kg/m^2^; (3) a history of hangover symptoms within the past month; (4) the ability to understand and comply with study procedures.

Exclusion criteria: (1) alcohol hypersensitivity or alcohol use disorder; (2) significant alcohol consumption (>21 drinks/week) within one week of the study; (3) the use of medications affecting alcohol metabolism within 30 days; (4) liver disease, gastrointestinal disorders, or other significant medical conditions; (5) pregnancy or lactation; (6) participation in other clinical trials within the past month.

### 2.3. Interventions

The test substance was provided in a 20 mL liquid sachet. The formulation contained 8.71 mL of java water-dropwort extract (equivalent to 6.69 g of raw plant material) as the primary active ingredient. The remaining volume consisted of a proprietary blend of pear concentrate, taurine, vitamin C, and milk thistle extract, formulated according to the manufacturer’s proprietary specifications for organoleptic enhancement and general nutritional support. The placebo (20 mL) was strictly designed to match the active formulation in visual appearance, viscosity, olfactory profile, and caloric density to ensure the preservation of the double-blind protocol. It consisted of purified water, fructooligosaccharide, and food-grade colorants and flavorings, completely devoid of the active botanical extracts. Both formulations were manufactured and provided by Jeonju Agro-biomaterial Research Institute.

### 2.4. Study Procedures

**Figure 1:**
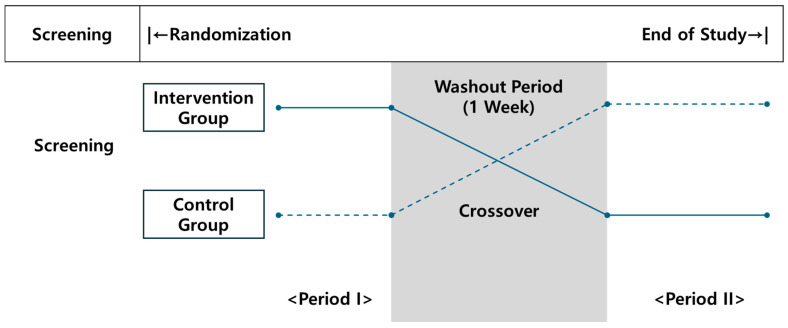
Overview of clinical trials.

The study followed a crossover design with two treatment periods separated by a 7-day washout period. Participants were randomly allocated to receive either the test formulation followed by placebo (Group A) or placebo followed by the test formulation (Group B) in a 1:1 ratio (Figure 1).

On each study visit, participants arrived fasting and consumed a standardized meal. Two hours after the meal, they received the assigned intervention. Thirty minutes later, participants consumed alcohol (20.1% soju, 0.8 g/kg body weight) within 30 min, accompanied by a small amount of a standardized snack. Water (200 mL) was provided four hours after alcohol consumption.

### 2.5. Outcome Measurements

**Primary endpoints**: Blood alcohol and acetaldehyde concentrations were measured at baseline (pre-alcohol), 0.5, 0.75, 1, 2, 4, 6, and 15 h post alcohol consumption. Blood samples were collected in EDTA tubes, and alcohol concentration was determined using an enzymatic assay. Acetaldehyde levels were measured using gas chromatography–mass spectrometry (GC-MS) at an external laboratory.

**Secondary endpoints**: Hangover symptoms were assessed using validated questionnaires: the Alcohol Hangover Severity Scale (AHSS) [14] at 6 h and the Acute Hangover Scale (AHS) [15] at 15 h post alcohol consumption.

**Safety assessments**: Vital signs, electrocardiogram, physical examination, and clinical laboratory tests were monitored. Adverse events were recorded throughout the study period.

### 2.6. Statistical Analysis

Statistical analyses were performed using SAS (version 9.4, SAS Institute Inc., Cary, NC, USA) and SPSS (version 26.0, IBM Corp., Armonk, NY, USA) software. The primary analysis population was per-protocol (PP), including participants who completed both treatment periods without major protocol violations. The paired t-test and Wilcoxon signed-rank test were used to compare outcomes between treatment groups. Area under the curve (AUC), maximum concentration (C_max_), and time to maximum concentration (T_max_) were calculated for alcohol and acetaldehyde concentrations. Statistical significance was set at *p* < 0.05.

## 3. Results

### 3.1. Enrollment

Of the 61 individuals who voluntarily signed informed consent forms and underwent screening, 25 were excluded due to failure to meet the inclusion/exclusion criteria (*n* = 17) or withdrawal of consent (*n* = 8). Consequently, 36 eligible participants were enrolled and randomized in a 1:1 ratio to Sequence A (test supplement followed by placebo; *n* = 18) or Sequence B (placebo followed by test; *n* = 18).

All 36 randomized participants received the allocated intervention and completed the follow-up in Phase 1. However, during the 7-day washout period following Phase 1, one participant in Sequence B withdrew consent and discontinued the study. As a result, 35 participants completed all procedures across both Phase 1 and Phase 2. The full analysis (FA) set was defined to include these 35 completers along with the data from the single dropout who completed Phase 1 (*n* = 36).

**Figure 2:**
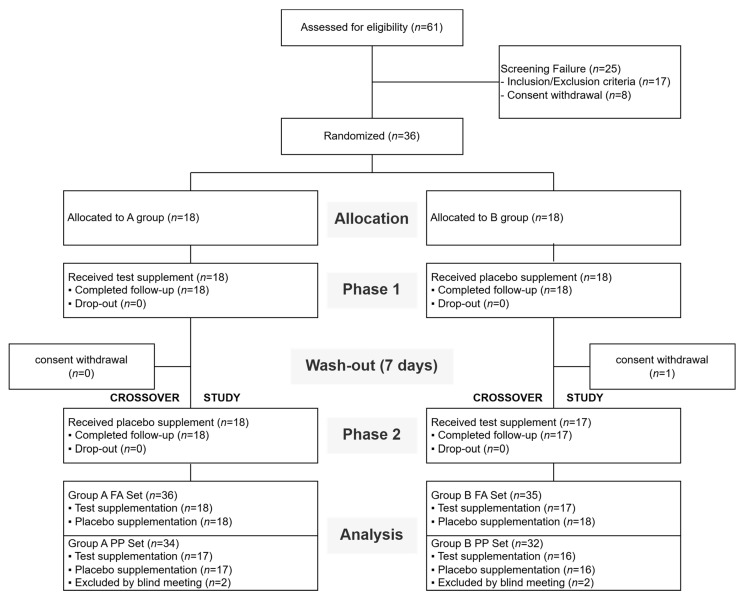
Flow diagram of participant recruitment, randomization, and study progression.

For the per-protocol (PP) analysis, a blind data review meeting was conducted prior to code breaking. During this review, two participants (one from each sequence) were excluded due to outlier values in blood acetaldehyde concentrations. Consequently, a total of 33 participants were included in the final PP analysis (Sequence A: *n* = 17; Sequence B: *n* = 16). The detailed flow of participants through the trial is summarized in Figure 2.

### 3.2. Participant Characteristics

The 36 enrolled participants exhibited favorable baseline profiles as detailed in Table 1, with 23 males (63.9%) and 13 females (36.1%) represented. Mean age was 23.92 ± 3.65 years, accompanied by an average body weight of 68.35 ± 12.83 kg and BMI of 23.46 ± 2.84 kg/m^2^. Systolic blood pressure averaged 125.08 ± 12.57 mmHg and diastolic 74.33 ± 11.30 mmHg, while smoking status and alcohol intake (1 unit = 10 g pure alcohol/week) were recorded at screening (Table 1).

**Table 1:** Baseline characteristics of study participants.

Variables	Total (*n* = 36)
Sex (M/F)	23/13
Age (years)	23.92 ± 3.65
Height (cm)	170.02 ± 9.06
Weight (kg)	68.35 ± 12.83
BMI (kg/m^2^)	23.46 ± 2.84
SBP (mmHg)	125.08 ± 12.57
DBP (mmHg)	74.33 ± 11.30
Pulse (beats/min)	76.00 ± 9.67
Temperature (°C)	36.50 ± 0.06
Smoking, *n* (%)	25 (30.56)
Smoking (cigarettes/day)	8.45 ± 3.56
Alcohol, *n* (%)	36 (100)
Quantity of Alcohol consumption (unit/week)	6.80 ± 4.30

Values are presented as mean ± standard deviation or number (%). Alcohol unit: 1 unit = 10 g pure alcohol.

### 3.3. Change in Blood Alcohol and Acetaldehyde Levels

**Figure 3:**
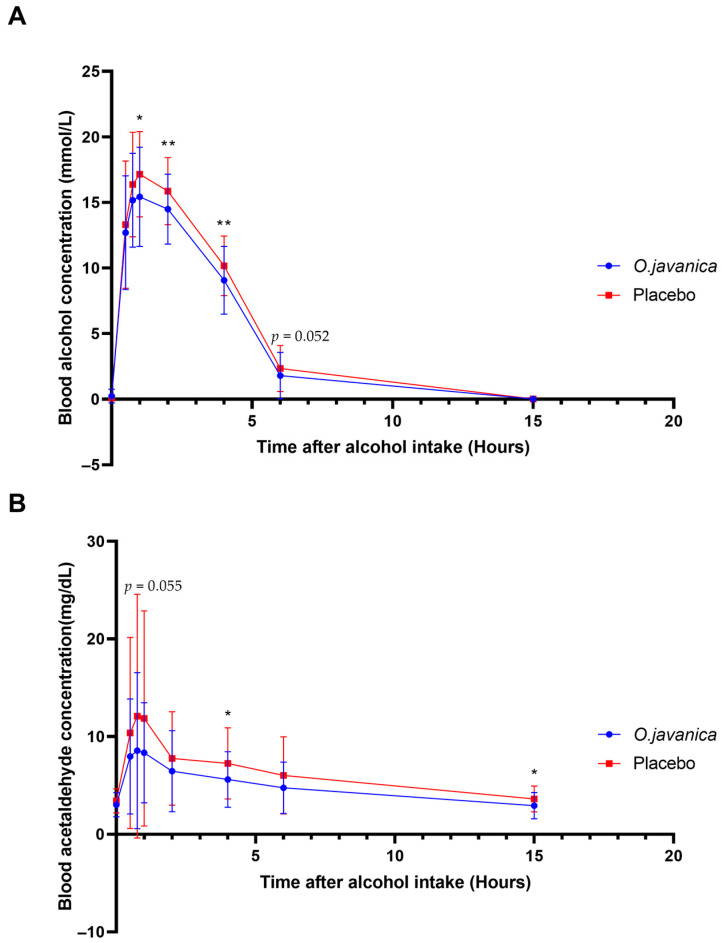
Effects of java water-dropwort (*O. javanica*) and placebo on blood alcohol and acetaldehyde concentrations after alcohol ingestion: (**A**) blood alcohol concentration; (**B**) blood acetaldehyde concentration. Data represent mean ± SD; data were compared between groups using paired *t*-test. * *p* < 0.05; ** *p* < 0.01.

**Figure 4:**
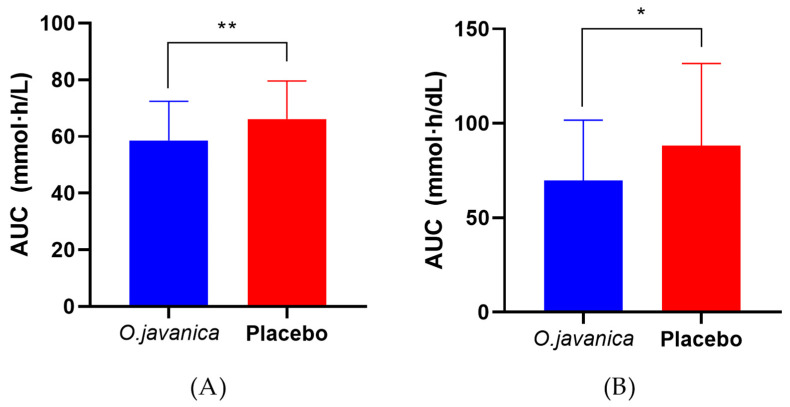
The effects of java water-dropwort (*O. javanica*) versus placebo on the area under the concentration–time curve (AUC) for blood alcohol and acetaldehyde following alcohol ingestion: (**A**) alcohol AUC (** *p* < 0.01); (**B**) acetaldehyde AUC (* *p* < 0.05). Values represent the mean ± SD; intergroup comparisons by paired *t*-test.

Blood Alcohol Concentrations: As shown in Figure 3A, the java water-dropwort extract group demonstrated significantly lower blood alcohol concentrations at 1 h (*p* = 0.031), 2 h (*p* = 0.028), and 4 h (*p* = 0.019) post alcohol consumption compared to the placebo group (*p* < 0.05). Furthermore, the blood alcohol AUC was significantly reduced in the java water-dropwort extract group (58.626 ± 13.868 mmol·h/L) compared to the placebo group (66.194 ± 13.465 mmol·h/L, *p* = 0.008) (Figure 4A). No significant differences were observed in C_max_ (16.626 ± 3.302 vs. 18.088 ± 3.018 mmol/L, *p* = 0.057) or T_max_ (1.023 ± 0.494 vs. 1.136 ± 0.556 h, *p* = 0.354) (Table 2).

Blood Acetaldehyde Concentrations: Acetaldehyde concentrations were significantly lower in the java water-dropwort extract group at 4 and 15 h post alcohol consumption (*p* < 0.05), as illustrated in Figure 3B. The acetaldehyde AUC was significantly reduced with java water-dropwort extract treatment (69.794 ± 31.866 mg·h/dL) compared to placebo (88.205 ± 43.482 mg·h/dL, *p* = 0.031) (Figure 4B). No significant differences were found in C_max_ (9.729 ± 8.170 vs. 13.422 ± 12.670 mg/dL, *p* = 0.074) or T_max_ (1.015 ± 0.827 vs. 1.424 ± 1.525 h, *p* = 0.211) (Table 3).

### 3.4. Subjective Hangover Symptoms

Hangover severity was assessed using the Alcohol Hangover Severity Scale (AHSS) and the Acute Hangover Scale (AHS). AHSS total scores showed a trend toward improvement in the java water-dropwort extract group (10.879 ± 10.222) compared to the placebo group (21.333 ± 20.938, *p* = 0.113), although this was not statistically significant. However, five individual AHSS symptoms showed significant improvements: sweating (*p* = 0.003), tremor (*p* = 0.034), gastrointestinal disturbances (*p* = 0.004), nausea (*p* = 0.003), and dizziness (*p* = 0.003) (Table 4).

AHS total scores were significantly lower in the java water-dropwort extract group (4.030 ± 2.640) compared to the placebo group (8.606 ± 8.291, *p* = 0.026). Five AHS individual items showed significant improvements: headache (*p* = 0.046), loss of appetite (*p* = 0.001), gastrointestinal disturbances (*p* = 0.023), nausea (*p* = 0.016), and heart palpitations (*p* = 0.031) (Table 5).

### 3.5. Safety

No adverse events or serious adverse events were reported during the study period. Vital signs, including systolic and diastolic blood pressure, pulse rate, and body temperature, showed no statistically significant differences between the test and placebo groups before and after administration (Table 6).

Regarding clinical laboratory tests, including hematology, blood biochemistry, and urinalysis, no participants showed clinically significant abnormalities. Although minor deviations from reference ranges were observed in some participants (e.g., RBC, CPK, ketones), these were determined by the principal investigator to be temporary fluctuations related to alcohol consumption, sleep deprivation, or hydration status, and were not clinically significant.

## 4. Discussion

This randomized, double-blind, placebo-controlled crossover trial demonstrated that java water-dropwort extract significantly enhanced alcohol metabolism and improved hangover symptoms in healthy adults. The primary findings include significant reductions in both blood alcohol and acetaldehyde AUCs, along with improved subjective hangover symptom scores.

The enhanced alcohol clearance observed with java water-dropwort extract treatment aligns with previous preclinical studies suggesting that bioactive compounds in java water-dropwort may stimulate alcohol-metabolizing enzymes [7,9]. The 11.4% reduction in blood alcohol AUC and 20.9% reduction in acetaldehyde AUC represent clinically meaningful improvements in alcohol metabolism. These findings are consistent with recent clinical trials investigating other natural compounds for hangover relief, which reported similar magnitudes of improvement in alcohol and acetaldehyde clearance [11,12,16,17].

A potential limitation of the present study lies in the utilization of a standardized 0.8 g/kg body weight alcohol challenge dose, which may diverge from naturalistic, real-world drinking behaviors. However, the implementation of this standardized dose is a methodological necessity strongly aligned with the consensus guidelines of the Alcohol Hangover Research Group (AHRG) [18]. Pharmacologically, a 0.8 g/kg dose is precisely calibrated to safely elevate peak blood alcohol concentrations to a threshold essential to reliably and uniformly induce measurable next-day hangover pathology across a cohort. Utilizing higher doses introduces severe ethical risks, including acute emesis, which inherently disrupts gastric absorption modeling and invalidates the area under the curve (AUC) measurements for blood ethanol and acetaldehyde.

The mechanisms underlying these effects likely involve multiple pathways. Java water-dropwort contains abundant phenolic compounds, including isorhamnetin, hyperoside, chlorogenic acid, caffeic acid, 5-O-caffeoylquinic acid, butanedioic acid, gallic acid, and persicarin, which have been shown to possess antioxidant and hepatoprotective properties [9,19]. Phytochemical profiling indicates that java water-dropwort is densely populated with bioactive flavonoids and phenolic compounds, specifically persicarin, isorhamnetin, and *p*-coumaric acid. Prior in vivo murine models have established that these specific constituents significantly enhance the expression and enzymatic efficiency of both ADH and ALDH, facilitating the accelerated systemic clearance of acetaldehyde [9,19]. Furthermore, the mitigation of acute hangover severity scores is likely linked to the extract’s robust antioxidant properties. The phenolic constituents of java water-dropwort are documented activators of the Nuclear Factor Erythroid 2-Related Factor 2 (Nrf2) signaling cascade. By promoting this pathway, the extract induces the transcription of critical endogenous antioxidant enzymes—such as superoxide dismutase (SOD), catalase (CAT), and glutathione peroxidase (GPx)—thereby neutralizing reactive oxygen species (ROS) generated during ethanol oxidation, suppressing systemic inflammatory cytokines, and actively relieving neuroinflammatory hangover symptoms [6,7,20].

These compounds may enhance the activity of ADH and ALDH enzymes, thereby accelerating alcohol metabolism and acetaldehyde clearance. Additionally, the formulation contained taurine, vitamin C, and milk thistle extract, which are known to support liver function and may contribute synergistically to the observed effects [21,22,23].

The improvement in hangover symptoms, as measured by validated questionnaires, provides clinical relevance to the biochemical findings. The significant reduction in AHS total scores and improvements in specific symptoms such as nausea, headache, and gastrointestinal disturbances suggest that the enhanced alcohol metabolism translates into meaningful symptom relief [15]. The fact that AHSS total scores showed a trend toward improvement, with significant improvements in several individual items, further supports the clinical efficacy of the intervention [14].

The crossover design of this study eliminated potential confounding factors related to individual variations in alcohol metabolism, as each participant served as their own control. The 7-day washout period was sufficient to eliminate carryover effects, and the randomization ensured balanced treatment sequences [16,24,25]. The use of validated hangover symptom scales and objective biochemical measurements strengthens the reliability of the findings.

Several limitations should be acknowledged. First, the study population was relatively young and healthy, which may limit the generalizability to older adults or individuals with comorbidities. Second, the alcohol challenge dose (0.8 g/kg body weight) may not reflect real-world drinking patterns, although it was standardized to ensure consistent exposure across participants. Third, the study duration was limited to acute effects, and long-term safety and efficacy were not evaluated.

The safety profile of java water-dropwort extract was excellent, with no adverse events reported. This finding is consistent with its traditional use as a food ingredient in East Asian cuisine and its generally recognized safety profile. The absence of significant changes in clinical laboratory parameters further supports the safety of short-term use.

Future research directions include investigating dose–response relationships, evaluating effects in different populations, and exploring the underlying molecular mechanisms. Long-term studies assessing the chronic use of java water-dropwort extract for individuals with frequent alcohol consumption may also be warranted.

## 5. Conclusions

Java water-dropwort (*O*. *javanica*) extract significantly enhanced alcohol metabolism by reducing blood alcohol and acetaldehyde concentrations and improved hangover symptoms in healthy adults. The intervention demonstrated an excellent safety profile with no reported adverse events. These findings suggest that java water-dropwort extract may serve as an effective natural intervention for alcohol-induced hangover management. However, the application of these findings is subject to distinct demographic limitations. The clinical cohort was restricted to young, healthy adults (aged 19–40 years). Extrapolating these results to older demographics characterized by age-related hepatic decline or to individuals with specific genetic polymorphisms in alcohol-metabolizing enzymes—such as the ALDH2*2 variant, which is highly prevalent in East Asian populations and severely impairs acetaldehyde clearance—requires further validation [26]. Subsequent large-scale, longitudinal clinical trials encompassing diverse age groups and varying genetic phenotypes are imperative to fully validate the broad-spectrum therapeutic utility and long-term safety of the extract.

## Figures and Tables

**Table 2:** Alcohol concentration in blood.

Variable	*O. javanica* Group (*n* = 33)	Placebo Group (*n* = 33)	*p*-Value
Alcohol concentration in blood (mmol/L)			
0 h	0.241 ± 0.513	0.084 ± 0.167	0.100
0.5 h	12.698 ± 4.335	13.302 ± 4.856	0.563
0.75 h	15.173 ± 3.583	16.371 ± 3.980	0.105
1 h	15.428 ± 3.776	17.149 ± 3.246	0.031
2 h	14.492 ± 2.667	15.863 ± 2.548	0.028
4 h	9.067 ± 2.585	10.167 ± 2.273	0.019
6 h	1.798 ± 1.770	2.342 ± 1.761	0.052
15 h	0.023 ± 0.051	0.024 ± 0.055	0.925
AUC (mmol·h/L)	58.626 ± 13.868	66.194 ± 13.465	0.008
C_max_ (mmol/L)	16.626 ± 3.302	18.088 ± 3.018	0.057
T_max_ (h)	1.023 ± 0.494	1.136 ± 0.556	0.354

Values are presented as mean ± SD. Analyzed using paired *t*-test (compared between groups).

**Table 3:** Acetaldehyde concentration in blood.

Variable	*O. javanica* Group (*n* = 33)	Placebo Group (*n* = 33)	*p*-Value
Acetaldehyde concentration in blood (mg/dL)			
0 h	3.021 ± 1.244	3.411 ± 1.228	0.055
0.5 h	7.957 ± 5.885	10.379 ± 9.784	0.204
0.75 h	8.564 ± 7.994	12.101 ± 12.482	0.085
1 h	8.340 ± 5.122	11.862 ± 11.012	0.055
2 h	6.463 ± 4.162	7.766 ± 4.783	0.170
4 h	5.610 ± 2.851	7.253 ± 3.639	0.025
6 h	4.759 ± 2.627	6.030 ± 3.961	0.086
15 h	2.930 ± 1.344	3.618 ± 1.330	0.023
AUC (mg·h/dL)	69.794 ± 31.866	88.205 ± 43.482	0.031
C_max_ (mg/dL)	9.729 ± 8.170	13.422 ± 12.670	0.074
T_max_ (h)	1.015 ± 0.827	1.424 ± 1.525	0.211

Values are presented as mean ± SD. Analyzed using paired *t*-test (compared between groups).

**Table 4:** Severity of Alcohol Hangover Symptoms (AHSs) questionnaire.

Item	*O. javanica* Group (*n* = 33)	Placebo Group (*n* = 33)	*p*-Value
Fatigue (being tired)	3.091 ± 2.296	4.000 ± 2.828	0.281
Apathy (lack of interest/concern)	0.758 ± 1.501	1.424 ± 2.136	0.159
Concentration problems	1.182 ± 1.776	2.273 ± 2.661	0.081
Clumsiness	0.848 ± 1.439	1.273 ± 2.183	0.472
Confusion	0.939 ± 1.676	1.485 ± 2.138	0.341
Thirst	1.727 ± 1.701	2.485 ± 2.266	0.285
Sweating	0.152 ± 0.364	0.848 ± 1.395	0.003
Shivering	0.303 ± 0.684	1.091 ± 2.082	0.034
Stomach pain	0.273 ± 0.761	1.394 ± 2.371	0.004
Nausea	0.515 ± 1.202	2.091 ± 2.909	0.003
Dizziness	0.606 ± 1.499	1.939 ± 2.657	0.003
Heart pounding	0.485 ± 1.349	1.030 ± 1.403	0.052
Total Score	10.879 ± 10.222	21.333 ± 20.938	0.113

Values are presented as mean ± SD. Each item was graded from 0 (symptom absent) to 10 (extremely severe symptom). Analyzed by Wilcoxon’s signed-rank test.

**Table 5:** Severity of Acute Hangover Symptoms (AHSs) questionnaire.

Item	*O. javanica* Group (*n* = 33)	Placebo Group (*n* = 33)	*p*-Value
Hangover	0.424 ± 0.708	0.606 ± 0.899	0.284
Thirsty	1.242 ± 0.969	2.061 ± 2.091	0.051
Tired	1.455 ± 1.092	1.939 ± 1.519	0.175
Headache	0.394 ± 0.827	1.091 ± 1.774	0.046
Dizziness, faintness	0.121 ± 0.331	0.455 ± 1.034	0.086
Loss of Appetite	0.030 ± 0.174	0.636 ± 1.055	0.001
Stomach ache	0.212 ± 0.545	0.818 ± 1.509	0.023
Nausea	0.121 ± 0.415	0.727 ± 1.442	0.016
Heart racing	0.030 ± 0.174	0.273 ± 0.626	0.031
Total Score	4.030 ± 2.640	8.606 ± 8.291	0.026

Values are presented as mean ± SD. Each item was graded from 0 (symptom absent) to 7 (extremely severe symptom). Analyzed by Wilcoxon’s signed-rank test.

**Table 6:** Comparison of vital signs between test and placebo groups.

Variable	Time Point	*O. javanica* Group (*n* = 35)	Placebo Group (*n* = 36)	*p*-Value
Systolic BP (mmHg)	Pre-ingestion	121.89 ± 12.28	121.89 ± 12.64	0.999
	Post-ingestion	120.34 ± 11.72	118.86 ± 11.39	0.591
Diastolic BP (mmHg)	Pre-ingestion	72.29 ± 9.31	72.25 ± 8.94	0.987
	Post-ingestion	74.09 ± 9.24	74.69 ± 9.79	0.788
Pulse rate (beats/min)	Pre-ingestion	82.23 ± 9.86	82.86 ± 10.95	0.799
	Post-ingestion	70.49 ± 9.95	71.97 ± 9.30	0.518
Body Temperature (°C)	Pre-ingestion	36.47 ± 0.12	36.50 ± 0.11	0.290
	Post-ingestion	36.48 ± 0.05	36.49 ± 0.04	0.736

Values are presented as mean ± SD. Analyzed by independent *t*-test (compared groups).

## Data Availability

The data presented in this study are available on request from the corresponding author. The data are not publicly available due to privacy and ethical restrictions.

## Abbreviations

The following abbreviations are used in this manuscript:

ADH	Alcohol Dehydrogenase
AHS	Acute Hangover Scale
AHSS	Alcohol Hangover Severity Scale
ALDH	Aldehyde Dehydrogenase
AUC	Area Under the Curve
BMI	Body Mass Index
BP	Blood Pressure
C_max_	Maximum Concentration
EDTA	Ethylenediaminetetraacetic Acid
FA	Full Analysis
GC-MS	Gas Chromatography–Mass Spectrometry
IRB	Institutional Review Board
PP	Per-Protocol
SD	Standard Deviation
T_max_	Time to Maximum Concentration

## References

[1] Risnita S., Iseric E., Zijlstra M.N., Stock A.K., Verster J.C. (2025). The Relationship Between Alcohol Hangover Frequency and Hangover Severity. J. Clin. Med..

[2] Rothman R., Hayley A.C., Aitken B., Downey L.A. (2026). A Systematic Review of the Impact of the Alcohol Hangover Upon Negative Affect. Drug Alcohol Rev..

[3] Severeijns N.R., Sips A.S.M., Merlo A., Bruce G., Verster J.C. (2024). Absenteeism, Presenteeism, and the Economic Costs of Alcohol Hangover in The Netherlands. Healthcare.

[4] Iseric E., Scholey A., Verster J.C. (2024). Alcohol hangovers as a predictor of the development of immune-related chronic diseases. Alcohol Clin. Exp. Res..

[5] Mackus M., Loo A.J.V., Garssen J., Kraneveld A.D., Scholey A., Verster J.C. (2020). The Role of Alcohol Metabolism in the Pathology of Alcohol Hangover. J. Clin. Med..

[6] Bae U.J., Jang H.N., Lee S.H., Kim J.Y., Kim G.C. (2022). Oenanthe javanica Ethanolic Extract Alleviates Inflammation and Modifies Gut Microbiota in Mice with DSS-Induced Colitis. Antioxidants.

[7] Gam D.H., Park J.H., Kim S.H., Kang M.H., Kim S.B., Kim J.W. (2022). Production of Bioactive Substances to Alleviates Hangover and Ethanol-Induced Liver Damage through Fermentation of Oenanthe javanica Using Lactiplantibacillus plantarum. Molecules.

[8] Lu C.L., Li X.F. (2019). A Review of Oenanthe javanica (Blume) DC. as Traditional Medicinal Plant and Its Therapeutic Potential. Evid.-Based Complement. Altern. Med..

[9] Kim J.Y., Kim K.H., Lee Y.J., Lee S.H., Park J.C., Nam D.H. (2009). Oenanthe javanica extract accelerates ethanol metabolism in ethanol-treated animals. BMB Rep..

[10] Lee D.H., Lee J.S., Lee I.H., Hong J.T. (2020). Therapeutic potency of fermented field water-dropwort (*Oenanthe javanica* (Blume) DC.) in ethanol-induced liver injury. RSC Adv..

[11] Jung S.H., Lee Y.H., Lee E.K., Park S.D., Shim J.J., Lee J.L., Yoo H.H. (2023). Effects of Plant-Based Extract Mixture on Alcohol Metabolism and Hangover Improvement in Humans: A Randomized, Double-Blind, Paralleled, Placebo-Controlled Clinical Trial. J. Clin. Med..

[12] Lee K.W., Xu G., Paik D.H., Shim Y.Y., Reaney M.J.T., Park I., Lee S.H., Park J.Y., Park E., Lee S.B. (2024). Clinical Evaluation of Hovenia dulcis Extract Combinations for Effective Hangover Relief in Humans. Foods.

[13] Ministry of Food and Drug Safety (2025). Regulation on Recognition of Functional Ingredient and Standard/Specification for Health Functional Foods (Article 14, Paragraph 8, Item C).

[14] Penning R., McKinney A., Bus L.D., Olivier B., Slot K., Verster J.C. (2013). Measurement of alcohol hangover severity: Development of the Alcohol Hangover Severity Scale (AHSS). Psychopharmacology.

[15] Rohsenow D.J., Howland J., Minsky S.J., Greece J., Almeida A., Roehrs T.A. (2007). The Acute Hangover Scale: A new measure of immediate hangover symptoms. Addict. Behav..

[16] Paik D.H., Lee K.W., Shim Y.Y., Reaney M.J.T., Park I., Lee S.H., Park J.Y., Park E., Lee S.B., Kim I.A. (2024). Efficacy of Hovenia dulcis Fruit Extract in Hangover Mitigation: Double-Blind Randomized Clinical Evaluation. Foods.

[17] Lee H.S., Isse T., Kawamoto T., Baik H.W., Park J.Y., Yang M. (2013). Effect of Korean pear (Pyruspyrifolia cv. Shingo) juice on hangover severity following alcohol consumption. Food Chem. Toxicol..

[18] Verster J.C., Stephens R., Penning R., Rohsenow D., McGeary J., Levy D., McKinney A., Finnigan F., Piasecki T.M., Adan A. (2010). The alcohol hangover research group consensus statement on best practice in alcohol hangover research. Curr. Drug Abus. Rev..

[19] Ai G., Huang Z.M., Liu Q.C., Han Y.Q., Chen X. (2016). The protective effect of total phenolics from Oenanthe Javanica on acute liver failure induced by D-galactosamine. J. Ethnopharmacol..

[20] He W.J., Lv C.H., Chen Z., Shi M., Zeng C.X., Hou D.X., Qin S. (2023). The Regulatory Effect of Phytochemicals on Chronic Diseases by Targeting Nrf2-ARE Signaling Pathway. Antioxidants.

[21] Lin C.J., Chiu C.C., Chen Y.C., Chen M.L., Hsu T.C., Tzang B.S. (2015). Taurine Attenuates Hepatic Inflammation in Chronic Alcohol-Fed Rats Through Inhibition of TLR4/MyD88 Signaling. J. Med. Food.

[22] Tighe S.P., Akhtar D., Iqbal U., Ahmed A. (2020). Chronic Liver Disease and Silymarin: A Biochemical and Clinical Review. J. Clin. Transl. Hepatol..

[23] Luo X., Zhang W., He Z., Yang H., Gao J., Wu P., Ma Z.F. (2021). Dietary Vitamin C Intake Is Associated With Improved Liver Function and Glucose Metabolism in Chinese Adults. Front. Nutr..

[24] Lv X., Lu Y., Ding G., Li X., Xu X., Zhang A., Song G. (2022). Hydrogen intake relieves alcohol consumption and hangover symptoms in healthy adults: A randomized and placebo-controlled crossover study. Am. J. Clin. Nutr..

[25] Lee M.H., Kwak J.H., Jeon G., Lee J.W., Seo J.H., Lee H.S., Lee J.H. (2014). Red ginseng relieves the effects of alcohol consumption and hangover symptoms in healthy men: A randomized crossover study. Food Funct..

[26] Tadokoro T., Oura K., Nakahara M., Fujita K., Tani J., Morishita A., Kobara H. (2025). Genetic Polymorphisms of ALDH2 and ADH1B in Alcohol-Induced Liver Injury: Molecular Mechanisms of Inflammation and Disease Progression in East Asian Populations. Int. J. Mol. Sci..

